# Aminolysis of Various Aliphatic Polyesters in a Form of Nanofibers and Films

**DOI:** 10.3390/polym11101669

**Published:** 2019-10-14

**Authors:** Oliwia Jeznach, Dorota Kolbuk, Paweł Sajkiewicz

**Affiliations:** Institute of Fundamental Technological Research, Polish Academy of Sciences, Pawinskiego 5B, 02-106 Warsaw, Poland; dorotakolbuk@gmail.com (D.K.); plsajkiewicz@gmail.com (P.S.)

**Keywords:** aminolysis, polyester, electrospinning, nanofibers, film, surface chemical modification

## Abstract

Surface functionalization of polymer scaffolds is a method used to improve interactions of materials with cells. A frequently used method for polyesters is aminolysis reaction, which introduces free amine groups on the surface. In this study, nanofibrous scaffolds and films of three different polyesters–polycaprolactone (PCL), poly(lactide-*co*-caprolactone) (PLCL), and poly(l-lactide) (PLLA) were subjected to this type of surface modification under the same conditions. Efficiency of aminolysis was evaluated on the basis of ninhydrin tests and ATR–FTIR spectroscopy. Also, impact of this treatment on the mechanical properties, crystallinity, and wettability of polyesters was compared and discussed from the perspective of aminolysis efficiency. It was shown that aminolysis is less efficient in the case of nanofibers, particularly for PCL nanofibers. Our hypothesis based on the fundamentals of classical high speed spinning process is that the lower efficiency of aminolysis in the case of nanofibers is associated with the radial distribution of crystallinity of electrospun fiber with more crystalline skin, strongly inhibiting the reaction. Moreover, the water contact angle results demonstrate that the effect of free amino groups on wettability is very different depending on the type and the form of polymer. The results of this study can help to understand fundamentals of aminolysis-based surface modification.

## 1. Introduction

The interactions between scaffolds and cells are determined primarily by surface properties of materials, such as roughness and topography, chemical structure and functional groups, types and density of electrical charges, balance between hydrophilicity and hydrophobicity, and surface free energy [1,2,3,4,5,6,7,8,9]. To date, many methods have been proposed to induce changes of the surface physico-chemical properties in a way that makes it attractive for a specific application [10]. According to the literature, common strategies of polymer surface modification for biological applications include: Plasma [11] and laser treatment [12], wet chemical methods such as aminolysis [13], hydrolysis [14], ion implantation [15], blending with hydrophilic pluronics, segregating at the surface [16], and various approaches of protein immobilization [17,18,19].

Electrospinning is a method that allows to obtain fibers with nano and sub-micro diameters from polymer solution. This technique is commonly used to produce fibrous scaffolds mimicking the extracellular matrix (ECM) (e.g., [20]). From a technical point of view, electrospinning is a relatively simple method; however, from a physical perspective, the process is quite complex, leading to the formation of non-equilibrium structures. Such structures are very different from the structures formed using classical methods, raising new problems; for instance, related to the structure/properties radial gradients, which can be essential from the perspective of surface modifications.

Aliphatic polyesters, such as polyesters–polycaprolactone (PCL), poly(lactide-*co*-caprolactone) (PLCL), and poly(l-lactide) (PLLA) belong to the class of polymers commonly used in biomedical applications, due to their biocompatibility and degradability in human body conditions. Their advantages also include easily-tunable physical and mechanical properties, as well as ease of processing. It is worth noting that aliphatic polyesters are excellent materials to enhance surface properties using chemical methods like aminolysis because of the presence of ester bonds in the polymer chain. These, provided defined conditions, undergo rupture easily, causing, in the case of aminolysis, the formation of hydroxyl and amino groups at the material surface. In such a case, only one of the amino groups is consumed during the chain scission at the polymer surface, being converted to an amide group, while the second amine group remains free. This method is based on simple treating of the polymer scaffold in diamine solution (e.g., 1,6-hexanediamine [21], 1,2-ethanediamine [22], Fmoc-PEG-diamine [23], etc.) and was previously considered for improving the dyeing process and moisture regain in the textile industry [24,25,26]. In tissue engineering, aminolysis of polyesters is usually applied as the final [14,27] or the intermediate step in the biomolecule immobilization process [28]. In many papers, surface modification with aminolysis reaction is reported for polycaprolactone [14,29,30,31,32,33,34,35]. A smaller number of studies is related to the surface functionalization of other polyesters, such as polylactide [36,37,38] and poly(lactide-*co*-caprolactone) [19,39,40,41]. Moreover, in the case of PLLA meshes, aminolysis is frequently used as a method of fiber fragmentation [42,43,44,45,46]. The aim of our paper is to explain the different efficiency of the aminolysis at the same processing conditions for various aliphatic polyesters in the form of nanofibers and films. In the literature, there is a lack of such comparison. In our opinion, considering the extensive use of aminolysis for surface modification of polyester materials, a systematic study of the reaction efficiency and its impact on material properties is necessary. The polyesters were chosen regarding the glass transition temperature, being related to molecular mobility, which is expected to be crucial from the point of view of the possible free amine recombination process. We hypothesize that the difference between the efficiency of aminolysis is due to the structure peculiarities in fibers and films, which result from the difference in formation (crystallization) conditions.

## 2. Materials and Methods

### 2.1. Materials

Three types of polymers were used for fabrication of nanofibers and films: Poly(caprolactone) (PCL) (Sigma-Aldrich (Saint Louis, MO, USA), Mw = 80,000 g/mol, Tg = −60 °C), poly(l-lactide) (PLLA) (PL49, Corbion (Amsterdam, The Netherlands,), inherent viscosity = 4.9 dL/g, Tg = 58 °C), and poly(l-lactide-*co*-caprolactone) (PLCL) 70:30 (Resomer^®^ LC703S, Evonik (Weiterstadt, Germany), inherent viscosity = 1.3–1.8 dL/g, Tg = 32–42 °C). Solvents—acetic acid (AA) (purity degree 99.5%), formic acid (FA) (purity degree ≥ 98%), and hexafluoroisopropanol (HFIP) (purity degree 98.5%) were purchased from Poch (Gliwice, Poland), Sigma-Aldrich (Saint Louis, MO, USA), and Iris Biotech GmbH (Marktredwitz, Germany), respectively. Ethylenediamine (purity degree 99.5%), isopropanol (purity degree 99.8%), ninhydrin (purity degree 99%), and ethanol (purity degree 99.8%) were purchased from Chempur (Piekary Śląskie, Poland). For PCL and PLLA, glass transition temperatures were determined experimentally. Glass transition temperature of PLCL was assumed as stated by the producer.

### 2.2. Fabrication of PCL, PLCL, and PLLA Nanofibrous Scaffolds and Films

Three polymer solutions were used for electrospinning: 15% *w*/*w* solution of PCL in mixed solution of acetic acid and formic acid at 9:1 ratio, 7% *w*/*w* PLCL solution in HFIP, and 3.5% *w*/*w* solution of PLLA in HFIP. The electrospinning equipment (Bioinicia, Valencia, Spain) was operated in a horizontal mode. Solutions were pumped through a stainless steel needle placed at a distance of 15 cm from the collector rotating at a speed of 300 rpm. The applied voltage and ambient conditions were chosen to form nonwovens with appropriate morphology. The positive voltage applied to the needle was 13–15 kV for PCL and PLCL, and 13 kV for PLLA. The collector was maintained at the electrical potential of −2 kV in the case of PCL and PLCL and 0 kV for PLLA. Temperature and humidity conditions during electrospinning process were 26 °C, 36% for PCL, 38 °C, 40% for PLCL, and 24 °C, 35% for PLLA, respectively. The films were solution cast from the same solutions as the electrospun nanofibers and air-dried under the hood at room temperature.

### 2.3. Aminolysis Treatment

Samples were immersed in 6% *w*/*v* solution of 1,2-ethandiamine (ED) in isopropanol (app. sample/diamine solution ratio: 1.5 mg/mL) at 30 °C for 5 and 15 min with shaking at 100 rpm. Then, samples were washed three times with copious amount of water, followed by vacuum drying overnight.

### 2.4. Ninhydrin Assay

Effectiveness of the aminolysis was evaluated from concentration of free amino groups using ninhydrin staining. For quantitative tests, samples were transferred to a glass vial and 0.5 mL of 2% *w*/*v* ninhydrin solution was added. Then, they were heated for 10 min using a hot plate at 100 °C. After that, 1 mL of isopropanol (for PLLA and PLCL) or isopropanol/dioxane 1/1 solution (for PCL) was added. Absorbance of the samples was measured at 570 nm. The amount of free amino groups was calculated on the basis of the absorbance calibration curve with known concentration of ED in isopropanol solution. For visualization of staining, samples were placed onto glass dish, wetted with 0.2% *w*/*v* solution of ninhydrin in ethanol using a Pasteur pipette, and incubated at 40 °C for 15 min.

### 2.5. ATR–FTIR

Surface molecular structure of samples was analyzed using attenuated total reflectance Fourier transform infrared (ATR–FTIR) spectrometer Bruker Vertex 70 (Mannheim, Germany), in absorbance mode, in 4000–400 cm^−1^ range. The resolution of measurement was 2 cm^−1^.

### 2.6. WAXS

Wide angle X-ray scattering (WAXS) was applied for analysis of the supermolecular structure. WAXS measurements were performed using Bruker D8 Discover diffractometer Mannheim, Germany) with CuKα radiation operated at a voltage of 40 kV, and a current of 20 mA. All measurements were performed in reflection mode, using Goebel optics for beam formation: A 0.6 mm slit and Soller collimator. Highly sensitive Lynx Eye 1-D silicon strip detector was used. The range of diffraction angle, 2θ, was between 5° and 35°, with a step of 0.01° and a time of data accumulation at angular point of 0.2 s. The “empty” scan without a sample was subtracted and the default function of subtracting background was applied. Then, the WAXS profiles were deconvoluted numerically using PeakFit software assuming Pearson VII and Gauss functions for the crystal diffraction peaks from the amorphous halo, respectively. The degree of crystallinity was determined as the ratio of the area of all the crystalline diffraction peaks to the overall area of the profile. The full width at half maximum (FWHM) was used in determination of the relative mean crystal size, L, without correction for instrumental broadening, from the Scherrer equation:

L = Kλ/*β*cos*θ* (1)

where K is the dimensionless Scherrer shape factor, assumed as 0.9, *β* is the line broadening at FWHD, λ is the X-rays wavelength, and *θ* is the Bragg angle.

### 2.7. Mechanical Testing

Mechanical properties were measured using uniaxial testing machine Lloyd EZ-50 (New York, USA) equipped with handles for thin and delicate samples with a 50 N load cell for nanofibrous scaffolds and 100 N for polymer films under cross-head speed of 5 mm/min. For each type of material, three 10 × 40 mm dry samples were used (10 × 25 mm—actual area of testing). Sample thickness was measured with a thickness gauge. Mechanical properties—Young’s modulus, stress at break, and strain at break were determined from stress–strain curves.

### 2.8. Water Contact Angle

The wettability of the matrices was determined by water contact angle measurements using goniometer Data Physics OCA 15EC (Filderstadt, Germany). The results are reported as mean value ± standard deviation evaluated on five repetitions.

## 3. Results

### 3.1. Ninhydrin Staining of Free Amino Groups

**Figure 1:**
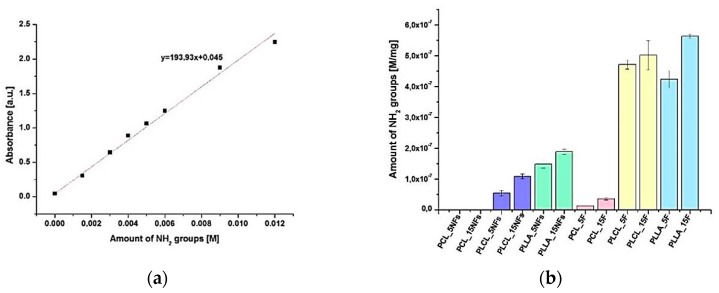
(**a**) Calibration curve used for amino groups quantification. (**b**) Amount of amino groups on modified nanofibers and films.

The amount of NH_2_ groups on the aminolyzed polymer nanofibers and films was measured by ninhydrin assay on the basis of previously obtained calibration curve (Figure 1). Presence of free amino groups was confirmed for all samples, except PCL nanofibers. This observation indicates that aminolysis at these conditions is not effective for PCL nanofibers, contrary to other investigated polyesters as well as to PCL films. This trend of more effective aminolysis for films as compared to nanofibers was also observed in results for PLLA and PLCL. Concentration of amino groups for films was considerably higher than for nanofibers—for example in the case of PLCL it reached 5.43 × 10^−8^ ± 9.0 × 10^−9^ M/mg for nanofibers and 4.72 × 10^−7^ ± 1.45 × 10^−8^ M/mg for film after 5 min of aminolysis. It is worth noting that effectiveness of the aminolysis was significantly lower for PCL film than for PLCL and PLLA film samples. Additionally, for each type of material, an increase in concentration of NH_2_ groups with reaction time was observed.

**Figure 2:**
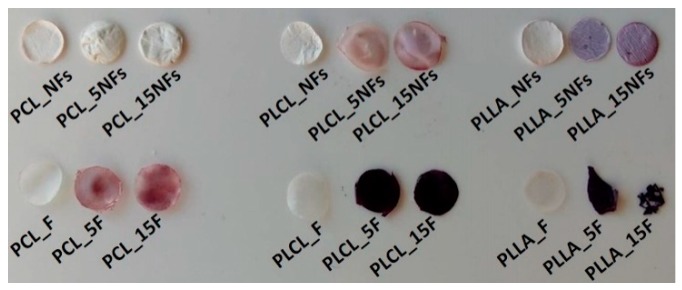
Representative images of samples after ninhydrin staining.

Figure 2 illustrates the qualitative results of ninhydrin staining. Among aminolyzed samples, only PCL nanofibers were unstained, what is in agreement with quantitative results (Figure 1b). In the case of PLLA film, highly effective aminolysis was accompanied by degradation, which is manifested by higher brittleness.

### 3.2. Molecular Structure from ATR–FTIR Analysis

**Figure 3:**
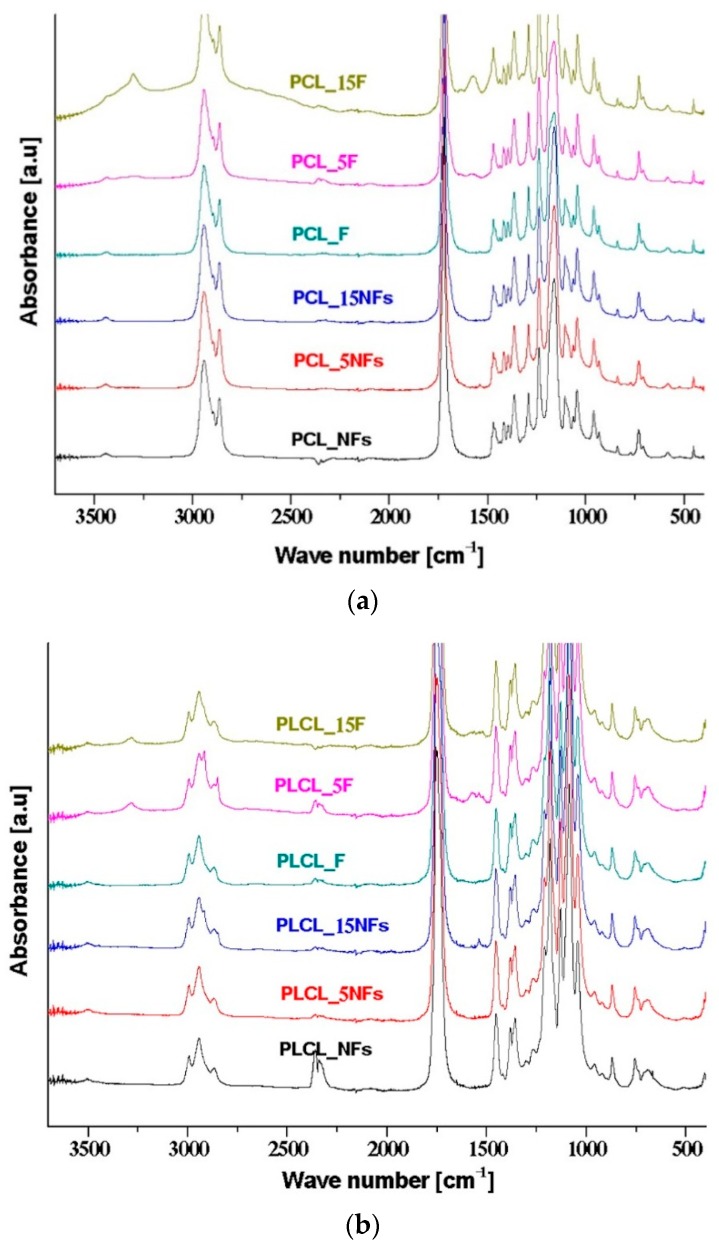

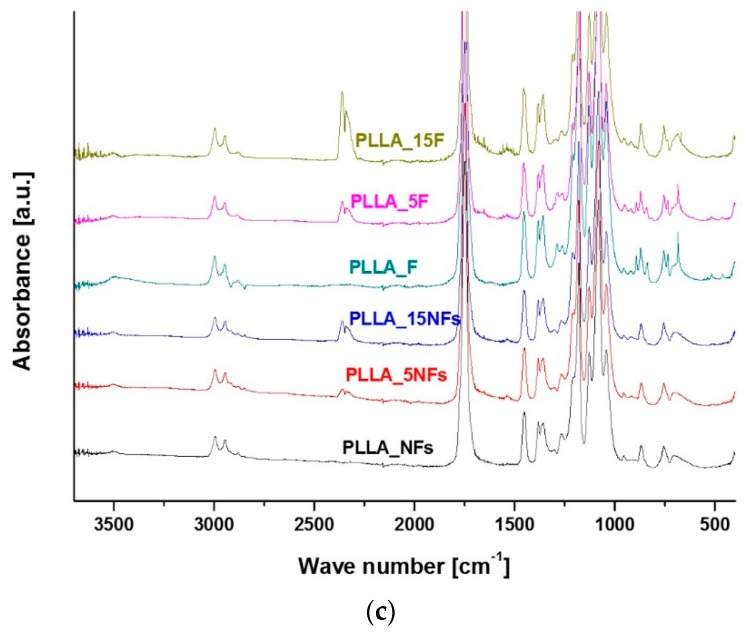
ATR–FTIR spectra of (**a**) PCL, (**b**) PLCL, and (**c**) PLLA nanofibers and films before and after the aminolysis treatment.

Figure 3 shows the ATR–FTIR spectra of PCL, PLCL, and PLLA nanofibers and films before and after the aminolysis. There are two amide bands resulting from nucleophilic attack of diamine on the carbonyl group, which may be treated as evidence of aminolysis reaction. The first band in the range 1510–1580 cm^−1^ is assigned to amide II, being mainly associated with N–H bending vibrations; the second one in the range 1600–1700 cm^−1^ corresponds to amide I, being mainly associated with C=O (carbonyl) stretching vibration (70–85%) and C–N group vibrations (10–20%). In the case of PCL (Figure 3a), the amide II band is observed for films after the treatment, which confirms the ninhydrin results, indicating the occurrence of aminolysis. Contrary to this, there are no amide peaks for PCL nanofibers after the aminolysis treatment, which is in agreement with ninhydrin staining results (Figure 2), indicating no aminolysis for PCL nanofibers. For modified PLCL films, broad peaks from the amide, mostly amide II bands, were observed (Figure 3b). In the case of PLCL nanofibers, the amide peaks were observed after 15 min of the treatment, only. The lack of amide peaks for PLCL nanofibers after 5 min of the treatment is most probably due to too low of a concentration of amine groups to be detected by the ATR–FTIR method. In the case of PLLA (Figure 3c), both amide I and II bands and for both nanofibers and films are observed, indicating that aminolysis is very effective for this polyester.

### 3.3. Supermolecular Structure (Crystallinity) from WAXS

**Figure 4:**
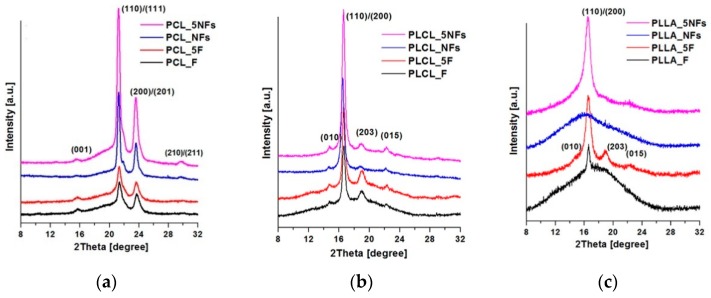
WAXS profile of casted films and fibers before and after the aminolysis treatment: (**a**) PCL, (**b**) PLCL, (**c**) PLLA.

**Figure 5:**
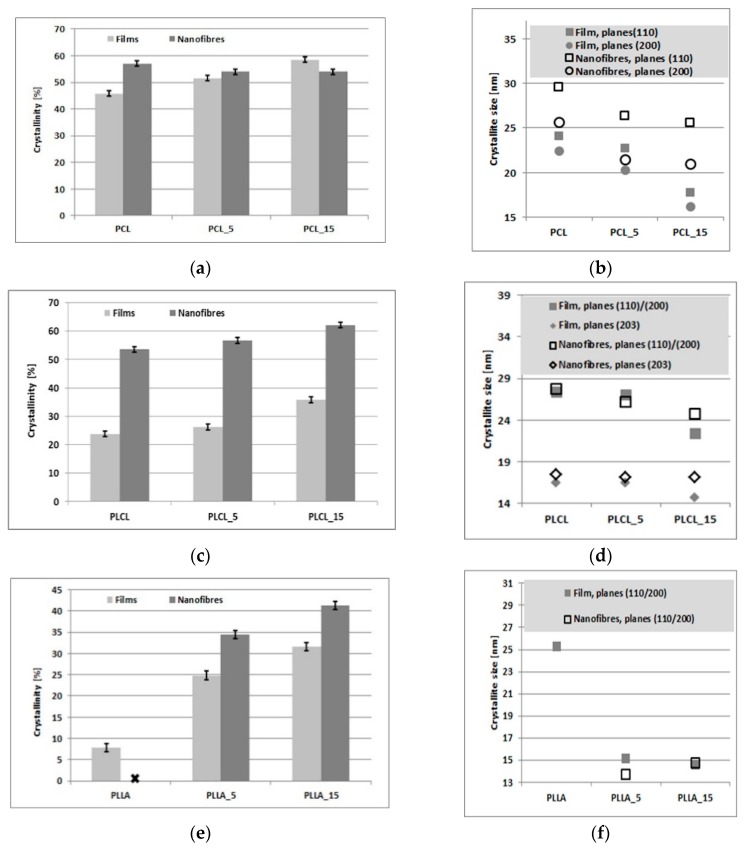
Crystallinity and crystal size of: (**a**,**b**) PCL, (**c**,**d**) PLCL, (**e**,**f**) PLLA.

WAXS profiles of PCL, PLLA, and PLCL films and nanofibers, before and after 5 min of the aminolysis treatment, are shown in Figure 4. Detailed information on the degree of crystallinity and crystal size/order are presented in Figure 5.

WAXS profiles of PCL indicate diffraction from crystal structure with strongest maximum at 2θ = 15.6 deg, 21.3 deg, 23.6 deg, and 29.7 deg corresponding to (001), (110)/(111), (200)/(201), and (210)/(201) lattice planes, respectively, and the maximum of the amorphous halo at 2θ = 21 deg (Figure 4a). This is in agreement with our previous studies [47,48].

Samples of PLCL show peaks at 2θ = 14.7 deg, 16.6 deg, 19 deg, and 22.4 deg (Figure 4b) corresponding to the (010), (110)/(200), (203), and (015) lattice planes, respectively [49].

The WAXS profile of PLLA is composed of a broad scattering peak from amorphous phase and small or zero intensity peaks from the crystal phase (Figure 4c). It may be seen that the aminolysis treatment leads to increase of PLLA crystallinity, making peaks at 2θ = 14.7 deg, 16.5 deg, 18.9 deg, and 22.3 deg, corresponding to the (010), (110)/(200), (203), and (015) lattice planes, evident [49,50].

The results shown in Figure 5 clearly indicate an increase of crystallinity with time of the aminolysis treatment for all polyesters films, as well as for PLLA and PLCL nanofibers. This increase in crystallinity is a result of molecular degradation leading to higher mobility of shorter molecules during the aminolysis treatment. The largest crystallinity increase is for PLLA and the lowest for PCL films, what is correlated with the ninhydrin results. No change in crystallinity is observed for treated PCL nanofibers (Figure 5a), for which the aminolysis treatment was found ineffective. A decrease of average crystal size with time of the treatment observed for PCL, PLCL, and PLLA nanofibers and films (Figure 5b,d,f) is most probably caused by formation of additional small crystals for most materials or disruption of existing crystals in the case of PCL nanofibers.

### 3.4. Mechanical Properties

**Figure 6:**
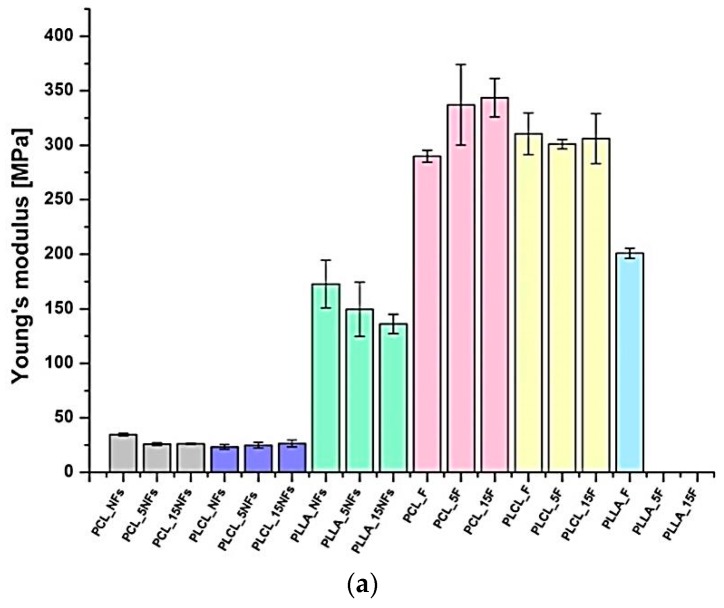

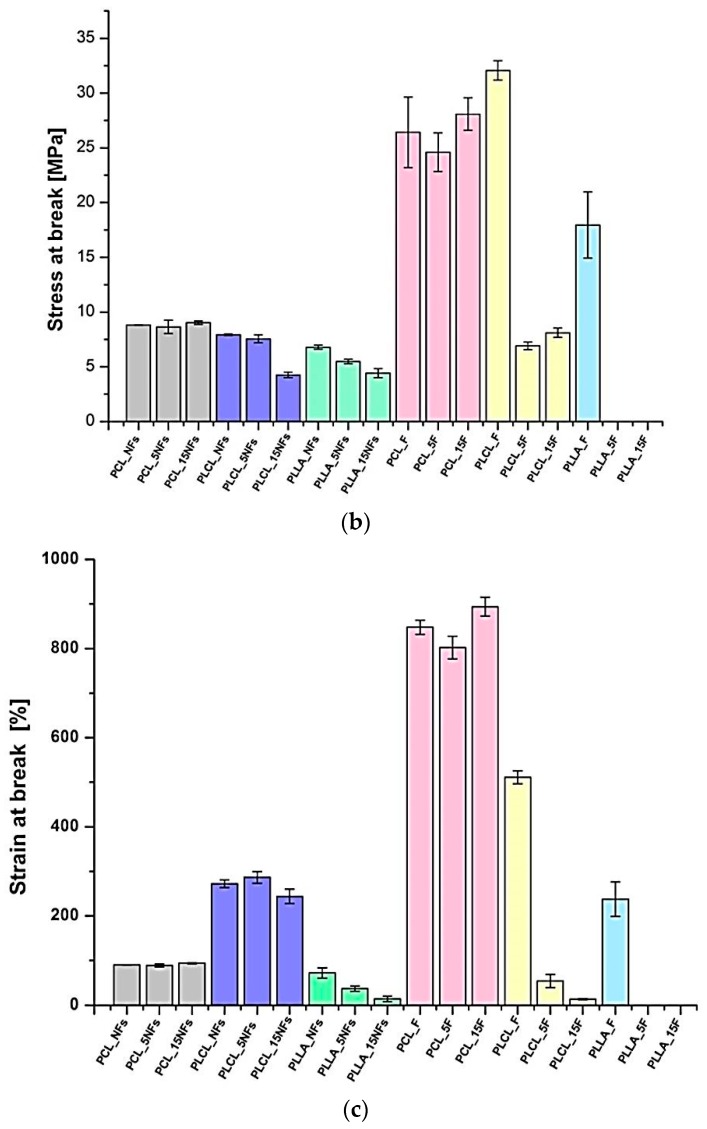
Mechanical properties of nanofibers and cast films before and after the aminolysis treatment: (**a**) Young’s modulus, (**b**) stress at break, (**c**) strain at break. For PLLA films after the treatment, there are no results because of complete degradation.

Figure 6 shows mechanical properties of samples before and after the aminolysis treatment. The changes of Young’s modulus (Figure 6a) were not informative from the perspective of aminolysis because of no simple correlation with crystallinity, as was expected. We anticipate that other factors like erosion of surface after the aminolysis treatment can influence the registered Young’s modulus. An increase in modulus with the treatment time correlated with increase in crystallinity was observed for PCL films, only. For other samples, no essential changes or even reduction of Young’s modulus with the treatment time was observed, with no correlation with crystallinity changes.

More direct information on the efficiency of the aminolysis can be drawn from analysis of the stress at break, which is molecular weight-dependent. An empirical equation, which combines the tensile strength, σ, with polymer molecular weight, has been proposed by Flory to predict variation of the stress at break with the polymer number–average molecular weight M_n_ [51]. This equation can be presented as:

σ = σ∞−B/M_n_, (2)

where σ∞ is the fracture strength at infinite molecular weight, and B is a constant derived from the relationship, discussed by Bersted and Anderson as:

B = Kσ∞M_c_, (3)

where K is a constant value between 2 and 3 and M_c_ is the critical molecular weight for entanglements [52].

Drop of the stress at break was noticed by us for all samples except PCL nanofibers and films, with a tendency to be more significant for longer times of the aminolysis treatment (Figure 6b). This stress at break reduction is a result of effective aminolysis, as evidenced directly by ninhydrin tests. The trends in the stress at break correspond with changes in the strain at break, with most visible reduction after the aminolysis treatment for PLLA nanofibers and PLCL films being as dramatic as from 511 ± 14.41% for PLCL_F to 13.6 ± 1.55% for PLCL_15F (Figure 6c).

### 3.5. Water Contact Angle

**Figure 7:**
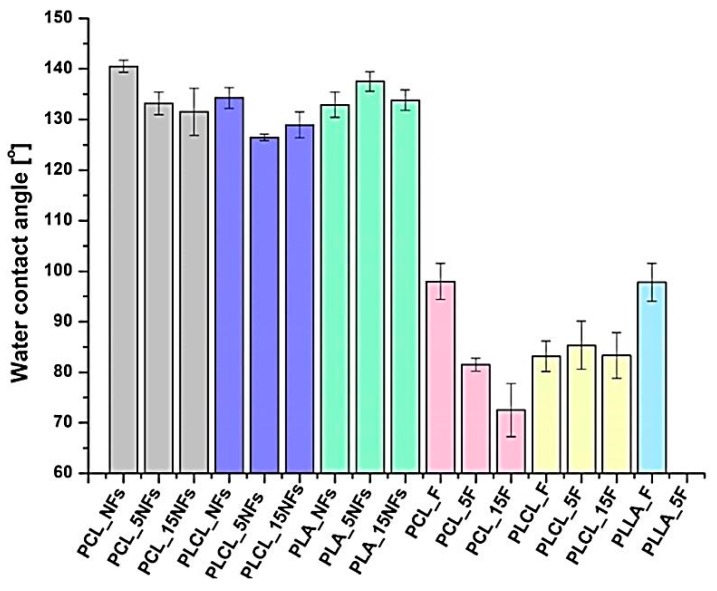
Water contact angle measurements for samples before and after the aminolysis treatment. For PLLA films after the treatment, there are no results because of complete degradation.

Figure 7 shows results of water contact angle (WCA) measurements. For PCL and PLCL nanofibers, a slight decrease in WCA after the aminolysis treatment was observed. In the case of PCL—from 140.55 ± 1.21° for pristine to 133.18 ± 2.21° for 5 min and 131.51 ± 4.67° for 15 min of the treatment. For PLCL nanofibers, the values of water contact angle were as follow: 134.27 ± 2.04°, 126.46 ± 0.64°, and 128.95 ± 2.55° for pristine and treated for 5 and 15 min samples, respectively. For PLLA nanofibers, 5 min of the treatment led to even an increase in WCA from 132.9 ± 2.48° for the pristine sample to 137.52 ± 1.92°. The general observation is that water contact angles are generally lower for film samples. After the treatment, a significant decrease of water contact angle was visible for PCL films. In the case of PLCL film, the values were almost unchanged. For PLLA films, it was impossible to measure water contact angle due to complete degradation.

## 4. Discussion

In this study we analyze the effects of the aminolysis treatment for three types of polyesters in the form of nanofibers and films.

The efficiency of the aminolysis for PCL, PLCL and PLLA samples was quantitatively measured by the amount of free amino groups using ninhydrin staining. The susceptibility of investigated polymer to aminolysis was the highest for PLLA, leading even to complete degradation of films, medium for PLCL, and the lowest for PCL. For example, after 5 min of the treatment, the amount of amino groups was about 35 times higher for PLCL in comparison with PCL film. As was expected, in the case of effective aminolysis treatment, the amount of free amino groups increased with the time of the treatment, which has already been reported [53,54]. The reactions under the same conditions turned out to be much more efficient in films as compared to nanofibers, despite the higher surface area to volume ratio for nanofibrous samples. The aminolysis treatment was ineffective for PCL nanofibers. We performed an additional experiment for PCL nanofibers with the aminolysis treatment at the same concentration and temperature conditions, but with the time extended to 144 h. However, morphology of nanofibers was unchanged and the amount of amine groups was only ca. 8.2 × 10^−8^ M/mg.

We provide additional results by the direct method of the ATR–FTIR technique and the indirect methods of WAXS and mechanical tensile tests. In the case of ATR–FTIR, the efficiency of the aminolysis treatment is confirmed by the appearance of the absorbance of the amide II and amide I bands.

The results of the ninhydrin tests are well correlated with the WAXS analysis of crystallinity. It is evident that crystallinity increases with the treatment time for all the samples, except PCL nanofibers. This crystallinity increase is due to chain scission, an effect of the ester bond cleavage resulting in higher molecular mobility allowing for additional crystallization. In the case of PCL nanofibers showing the aminolysis treatment as ineffective, there is also no increase in crystallinity as measured by WAXS. A reduction in the average crystal size with the treatment time observed for PCL, PLLA, and PLCL nanofibers and films is most probably a result of either formation of additional small crystals, in the case of the effective aminolysis for most materials, or disruption of existing crystals, in the case of PCL nanofibers.

The confirmation of the results of ninhydrin staining by the results of mechanical tensile tests is based on the molecular degradation, which accompanies the aminolysis process. Chain scission causes a decrease of the stress and strain at break. It is a well-known fact that reduction of chain length via aminolysis results in lower resistance against tension [55]. In our study, this process seems to be most intense in the case of PLLA nanofibers and films, as well as PLCL films. In the literature, there are many examples of reduction in mechanical properties, such as tensile strength, elongation at break, and even Young’s modulus, as a result of aminolysis process [14,56,57]. It should be noted that there are also studies in which authors observed an increase of tensile strength or elastic modulus after reaction [32]. In our work, we also noticed a small increase of mechanical properties for PCL films after the aminolysis treatment. This phenomenon was explained by Ganjalinia et al. as formation of cross-links between hydroxyl molecular chains of polymer and NH₂-terminated groups of reactant [55]. Summarizing the problem of various susceptibility of investigated polyesters to aminolysis, we can conclude that it is most probably associated with:

- Various ratios of ester to alkyl groups being the lowest for PCL and the highest for PLLA.
- Different crystallinity, being the highest for PCL and the lowest for PLLA. It is known that the availability of ester groups for aminolysis depends on the supermolecular organization, being lower for the crystal phase than for the amorphous one.
- Different rates of NH_2_ recombination after aminolysis, being dependent on the position of the aminolysis temperature in relation to glass transition temperature. It is anticipated that in the case of PCL with glass transition temperature far below aminolysis temperature, the free amine recombination rate should be much higher than for PLCL with T_g_ = 32–42 °C, and particularly for PLLA, for which aminolysis is performed below glass transition temperature.

The non-trivial problem is related to the effect of the form of material on the aminolysis effectiveness. Our results clearly indicate that aminolysis reaction turned out to be much easier in the case of films. Our hypothesis is that the reason is related to the radial distribution of structure in the case of nanofibers with more crystalline skin, which inhibits the reaction. There are some works regarding the fundamentals of polymer aminolysis, where it is claimed that aminolysis is a highly selective process, attacking primarily ester bonds in the amorphous regions, which is detected as a rapid drop in polymer molar mass and weight [26,47,58]. The less intensive attack of crystallites occurs at the further stage of the reaction. Considering the large differences in the effectiveness of aminolysis of film and electrospun nanofibers, we should return to the basics of fiber formation in classical processes of spinning from solution and melt. Relatively thick fibers formed in classical spinning processes allow to investigate experimentally the details of the internal structure, including its radial distribution. It was shown theoretically [59,60] that the crystallization driving force is higher at the fiber surface due to two reasons. The first one being of a thermodynamic nature is related to lower temperature at the jet surface in the case of melt spinning, or to a higher polymer concentration caused by surface solvent evaporation at the jet surface in the case of solution spinning. In both cases there is higher thermodynamic force at the jet surface because of higher supercooling or supersaturation. The second reason for the higher crystallization driving force at the jet surface is related to higher stress because of higher solution viscosity caused by lower temperature or higher polymer concentration. It leads to more effective molecular orientation at the surface, resulting in faster crystallization. Both factors are responsible for higher crystallinity at the fiber surface, as evidenced experimentally [61,62]. This radial distribution of structure with higher crystallinity at the surface compared to that at the core is narrower the higher the spinning rate [53]. For instance, the crystallinity of PET fibers spun at 5400 m/min and analyzed directly using localized electron scattering show well-pronounced crystallinity near the fiber surface with a practically amorphous fiber core [55]. This direct crystallinity analysis corresponds to measurements of radial variation of birefringence and Lorentz density [55]. It should be taken into account that the typical velocity of the jet during electrospinning, as measured directly using a moving substrate and high-speed camera, as well as laser Doppler velocimetry, is between 300 and 900 m/min [63,64,65], which is comparable to traditional dry spinning. Estimations of the electrospinning velocity based on the mass of the collected nanofibers range from 6000 m/min to an astonishing 60,000 m/min [66,67,68,69,70], so is the same order or even higher as for the processes of high speed spinning. However, it should be made aware that the estimation of the velocity from the mass of the collected nanofibers can be loaded with an error due to the splitting of the electrospinning jet or multiple electrospinning jets from the spinneret [71]. So, it is expected that in the case of solution electrospinning, there will be a similar rate of solvent evaporation resulting in similar crystallinity radial distribution in both processes, high speed spinning and electrospinning, allowing to transfer the conclusions from the high-speed spinning process to electrospinning. Some authors use the term skin/core morphology for this kind of structure radial distribution of fibers. In the case of electrospinning, there is no experimental evidence of such radial distribution of crystallinity because of extremely small diameter of fibers compared to those formed by classical spinning. There are only speculations using some experimental results on the possibility of the existence of the skin–core morphology, for instance in electrospun nylon-6 fibers [72]. Summarizing the problem, it is expected that the crystallinity of skin is much higher than for core of fibers, forming an effective barrier for aminolysis processing of nanofibers. In the case of casted films, we do not expect such gradient of crystallinity across the film thickness because of a much slower evaporation rate. An extreme example is the case of PCL nanofibers, for which aminolysis reaction is completely inefficient at given conditions.

The needs of harder reaction conditions for PCL nanofibers than for membranes was discussed by Piai et al. [73], who associated the problem with the high hydrophobicity of PCL nonwovens. However, our results show that the PLCL and PLLA nanofibers, which have very similar water contact angles to the PCL, are reactive at the same conditions, indicating that this is no problem of hydrophobicity. Our WAXS crystallinity measurements before aminolysis clearly show that bulk crystallinity of nanofibers is considerably higher than that of films, reaching 57% for PCL which is ca. 10% higher compared to PCL films. In the case of PLCL and PLLA nanofibers, crystallinity before aminolysis is lower compared to PCL, reaching 53%, while PLLA nanofibers remain practically amorphous. This very high bulk crystallinity of PCL nanofibers, together with highly probable crystallinity radial distribution, lets us suppose that surface PCL nanofibers’ crystallinity is extremely high, forming a strong barrier against aminolysis reaction.

According to the literature, additional information about aminolysis comes from water contact angle measurements, because introducing hydrophilic free amino groups on the surface should result in enhancement of wettability. Indeed, in the case of PCL film, we observed a significant decrease of water contact angle for modified samples, and the value was lower as time increased (25° drop for 15 min of aminolysis). Surprisingly, we also observed a slight decrease for PCL nanofibers, for which we did not detect any amino groups on the surface. However, this effect could be associated with the change of roughness after immersing in ED/isopropanol solution or surface hydrophilization with alcohol [74]. For the PLCL nanofibers, the observed decrease was also very slight being up to 9 degrees. Moreover, for the PLLA nanofibers and PLCL film, we did not observe any enhancement of wettability, or even a slight increase of water contact angle. Despite commonly emphasized improvement of wettability after aminolysis [75,76], this phenomenon seems to be much more complex. According to the literature, frequently only a slight decrease of water contact angle is observed [14,50,77]. Monnier et al. reported for PLLA film a similar observation to ours, for which water contact angle slightly increased with the time of aminolysis [78]. The authors proposed two mechanisms, which could be responsible for this phenomenon. Firstly, they proved appearing of double grafting during aminolysis, which means that two amine groups of diamine can react with ester bonds of polymer and, in this case, there is no free hydrophilic amino group. Indeed, in the literature there are reports of using diamines for cross-linking of polyester macromolecules [79]. According to the authors, another cause of contact angle increase could be related to the effect of hydrophobic alkyl chains of diamine, which can dominate the impact of free NH_2_ groups. A similar explanation was presented in the work of Bakry et al. [50], where identical values of advancing contact angle for pristine and aminolyzed PLLA film were assigned to re-orientation of the hydrophobic parts of grafted amine.

## 5. Conclusions

In these studies, we analyzed—using various methods, both direct and indirect—the efficiency of aminolysis under the same processing conditions for various aliphatic polyesters (PCL, PLCL, and PLLA) in the form of nanofibers and films. Considering the type of polymer, the order of aminolysis efficiency from highest to lowest is as follows: PLLA, PLCL, and PCL. Our explanation of this sequence of polymer susceptibility to aminolysis is related to different ratios of ester to alkyl groups, primary crystallinity, and NH_2_ recombination rate. Taking into account the form of the material, aminolysis reaction turned out to be much easier in the case of films. Our hypothesis is that the reason is related to the radial distribution of the structure, particularly of the supermolecular structure, with more crystalline skin in the case of nanofibers, strongly inhibiting the reaction. Our results of the amine group concentration analysis correspond to a change of crystallinity and mechanical properties, especially stress and strain at break after the aminolysis process. These results provide an important basis for the future research on the use of aminolysis for surface functionalization of polymers.

## References

[1] Ma Z., Mao Z., Gao C. (2007). Surface modification and property analysis of biomedical polymers used for tissue engineering. Colloid Surf. B Biointerfaces.

[2] Vasita R., Shanmugam I.K., Katt D.S. (2008). Improved Biomaterials for Tissue Engineering Applications: Surface Modification of Polymers. Curr. Top. Med. Chem..

[3] Wang Y.X., Robertson J.L., Spillman W.B., Claus R.O. (2004). Effects of the Chemical Structure and the Surface Properties of Polymeric Biomaterials on Their Biocompatibility. Pharm. Res..

[4] Budnicka M., Szymaniak M., Kołbuk D., Ruśkowski P., Gadomska-Gajadhur A. (2019). Biomineralization of poly-l-lactide spongy bone scaffolds obtained by freeze-extraction method. J. Biomed. Mater. Res. Part B Appl. Biomater..

[5] Kołbuk D., Urbanek O., Denis P., Choińska E. (2019). Sonochemical coating as an effective method of polymeric nonwovens functionalization. J. Biomed. Mater. Res. Part A.

[6] Jeznach O., Gajc M., Korzeb K., Kłos A., Orliński K., Stępień R., Krok-Borkowicz M., Rumian Ł., Pietryga K., Reczyńska K. (2018). New calcium-free Na_2_O-Al_2_O_3_-P_2_O_5_ bioactive glasses with potential applications in bone tissue engineering. J. Am. Ceram. Soc..

[7] Heljak M.K., Moczulska-Heljak M., Choińska E., Chlanda A., Kosik-Kozioł A., Jaroszewicz T., Jaroszewicz J., Swieszkowski W. (2018). Micro and nanoscale characterization of poly(DL-lactic-co-glycolic acid) films subjected to the L929 cells and the cyclic mechanical load. Micron.

[8] Ju J., Peng X., Huang K., Li L., Liu X., Chitrakar C., Chang L., Gu Z., Kuang T. (2019). High-performance porous PLLA-based scaffolds for bone tissue engineering: Preparation, characterization, and in vitro and in vivo evaluation. Polymer.

[9] Lin J., Zhou W., Han S., Bunpetch V., Zhao K., Liu C., Yin Z., Ouyang H. (2018). Cell-material interactions in tendon tissue engineering. Acta Biomater..

[10] Neděla O., Slepička P., Švorčík V. (2017). Surface Modification of Polymer Substrates for Biomedical Applications. Materials.

[11] Kooshki H., Ghollasi M., Halabian R., Kazemi N.M. (2019). Osteogenic differentiation of preconditioned bone marrow mesenchymal stem cells with lipopolysaccharide on modified poly-l-lactic-acid nanofibers. J. Cell. Physiol..

[12] Wismayer K., Mehrban N., Bowen J., Birchall M. (2019). Improving cellular migration in tissue-engineered laryngeal scaffolds. J. Laryngol. Otol..

[13] Zhu Y., Mao Z., Gao C. (2013). Aminolysis-based surface modification of polyesters for biomedical applications. RSC Adv..

[14] De Luca A.C., Terenghi G., Downes S. (2014). Chemical surface modification of poly-ε-caprolactone improves Schwann cell proliferation for peripheral nerve repair. J. Tissue Eng. Regen. Med..

[15] Chen X., Zhang X., Zhu Y., Zhang J., Hu P. (2003). Surface modification of polyhydroxyalkanoates by ion implantation. Characterization and cytocompatibility improvement. Polym. J..

[16] Birhanu G., Akbari Javar H., Seyedjafari E., Zandi-Karimi A., Dusti Telgerd M. (2018). An improved surface for enhanced stem cell proliferation and osteogenic differentiation using electrospun composite PLLA/P123 scaffold. Artif. Cells Nanomed. Biotechnol..

[17] Sadeghi A.R., Nokhasteh S., Molavi A.M., Khorsand-Ghayeni M., Naderi-Meshkin H., Mahdizadeh A. (2016). Surface modification of electrospun PLGA scaffold with collagen for bioengineered skin substitutes. Mater. Sci Eng. C Mater. Biol. Appl..

[18] Khademi F., Ai J., Soleimani M., Verdi J., Mohammad Tavangar S., Sadroddiny E., Massumi M., Mahmoud Hashemi S. (2017). Improved human endometrial stem cells differentiation into functional hepatocyte-like cells on a glycosaminoglycan/collagen-grafted polyethersulfone nanofibrous scaffold. J. Biomed. Mater. Res. B.

[19] Pan H., Zheng Q., Yang S., Guo X. (2014). Effects of functionalization of PLGA-[Asp-PEG]n copolymer surfaces with Arg-Gly-Asp peptides, hydroxyapatite nanoparticles, and BMP-2-derived peptides on cell behavior in vitro. J. Biomed. Mater. Res. A.

[20] Jun I., Han H.S., Edwards J.R., Jeon H. (2018). Electrospun Fibrous Scaffolds for Tissue Engineering: Viewpoints on Architecture and Fabrication. Int. J. Mol. Sci..

[21] Krithica N., Natarajan V., Madhan B., Sehgal P.K., Mandal A.B. (2012). Type I Collagen Immobilized Poly(caprolactone) Nanofibers: Characterization of Surface Modification and Growth of Fibroblasts. Adv. Eng. Mater..

[22] Cao L., Yu Y., Wang J., Werkmeister J.A., McLean K.M., Liu C. (2016). 2-N, 6-O-sulfated chitosan-assisted BMP-2 immobilization of PCL scaffolds for enhanced osteoinduction. Mater. Sci. Eng. C Mater. Biol. Appl..

[23] Hsieh Y.F., Sahagian K., Huang F., Xu K., Patel S., Li S. (2017). Comparison of plasma and chemical modifications of poly-L-lactide-co-caprolactone scaffolds for heparin conjugation. Biomed. Mater..

[24] Liu K., Chen L., Huang L., Lai Y. (2016). Evaluation of ethylenediamine-modified nanofibrillated cellulose/chitosan composites on adsorption of cationic and anionic dyes from aqueous solution. Carbohydr. Polym..

[25] Ohe T., Yoshimura Y. (2012). Reaction of PET fibers with ethylenediamine in a water solution containing surfactans. Sen’i Gakkaishi.

[26] Fukatsu K. (1992). Mechanical Properties of Poly(ethylene terephtalate) Fibers Imparted Hydrophilicity with Aminolysis. J. Appl. Polym. Sci..

[27] Zhao Y., Tan K., Zhou Y., Ye Z., Tan W.S. (2016). A combinatorial variation in surface chemistry and pore size of three-dimensional porous poly(ε-caprolactone) scaffolds modulates the behaviors of mesenchymal stem cells. Mater. Sci. Eng. C Mater. Biol. Appl..

[28] Zhu Y., Gao C., Liu X., He T., Shen J. (2004). Immobilization of Biomacromolecules onto Aminolyzed Poly(L-lactic acid) toward Acceleration of Endothelium Regeneration. Tissue Eng..

[29] Aguirre-Chagala Y.E., Altuzar V., León-Sarabia E., Tinoco-Magaña J.C., Yañez-Limón J.M., Mendoza-Barrera C. (2017). Physicochemical properties of polycaprolactone/collagen/elastin nanofibers fabricated by electrospinning. Mater. Sci. Eng. C Mater. Biol. Appl..

[30] Amirian J., Lee S.Y., Lee B.T. (2016). Designing of Combined Nano and Microfiber Network by Immobilization of Oxidized Cellulose Nanofiber on Polycaprolactone Fibrous Scaffold. J. Biomed. Nanotechnol..

[31] Song M.-J., Amirian J., Linh N.T.B., Lee B.-T. (2017). Bone morphogenetic protein-2 immobilization on porous. PCL-BCP-Col composite scaffolds for bone tissue engineering. J. Appl. Polym. Sci..

[32] Bhattacharjee P., Naskar D., Kim H.-W., Maiti T.K., Bhattacharya D., Kundu S.C. (2015). Non-mulberry silk fibroin grafted PCL nanofibrous scaffold: Promising ECM for bone tissue engineering. Eur. Polym. J..

[33] Kosmala A., Fitzgerald M.Z., Moore E.C., Stam F. (2017). Evaluation of a Gelatin Modified Poly(ɛ-Caprolactone) Film as a Scaffold for Lung Disease. Anal. Lett..

[34] Patel J.J., Flanagan C.L., Hollister S.J. (2015). Bone Morphogenetic Protein-2 Adsorption onto Poly-e-caprolactone Better Preserves Bioactivity In Vitro and Produces More Bone In Vivo than Conjugation Under Clinically Relevant Loading Scenarios. Tissue Eng. Part C Methods.

[35] Stevens J.S., De Luca A.C., Downes S., Terenghi G., Schroeder S.L.M. (2014). Immobilisation of cell-binding peptides on poly-ε-caprolactone (PCL) films: A comparative XPS study of two chemical surface functionalisation methods. Surf. Interface Anal..

[36] Pellegrino L., Cocchiola R., Francolini I., Lopreiato M., Piozzi A., Zanoni R., Scotto d’Abusco A., Martinelli A. (2017). Taurine grafting and collagen adsorption on PLLA films improve human primary chondrocyte adhesion and growth. Colloids Surf. B Biointerfaces.

[37] Xu F.J., Yang X.C., Li C.Y., Yang W.T. (2011). Functionalized Polylactide Film Surfaces via Surface-Initiated ATRP. Macromolecules.

[38] Zhang K., Zheng H., Liang S., Gao C. (2016). Aligned PLLA Nanofibrous Scaffolds Coated with Graphene Oxide for promoting neural cell growth. Acta Biomater..

[39] Li C., Wang L., Yang Z., Kim G., Chen H., Ge Z. (2012). A Viscoelastic Chitosan-Modified Three-Dimensional Porous Poly(L-lactide-co-ε-caprolactone) scaffold for cartilage tissue engineering. J. Biomater. Sci. Polym. Ed..

[40] Zhu Y., Chian K.S., Chan-Park M.B., Mhaisalkar P.S., Ratner B.D. (2006). Protein bonding on biodegradable poly(L-lactide-co-caprolactone). Biomaterials.

[41] Zhu Y., Leong M.F., Ong W.F., Chan-Park M.B., Chian K.S. (2007). Esophageal epithelium regeneration on fibronectin grafted poly(L-lactide-co-caprolactone) (PLLC) nanofiber scaffold. Biomaterials.

[42] Ahmad T., Lee J., Shin Y.M., Shin H.J., Madhurakat Perikamana S.K., Park S.H., Kim S.W., Shin H. (2017). Hybrid-spheroids incorporating ECM like engineered fragmented fibers potentiate stem cell function by improved cell/cell and cell/ECM. Acta Biomater..

[43] Castro A.G., Lo Giudice M.C., Vermonden T., Leeuwenburgh S.C., Jansen J.A., van den Beucken J.J., Yang F. (2016). A Top-Down Approach for the Preparation of Highly Porous PLLA Microcylinders. ACS Biomater. Sci. Eng..

[44] Castro A.G.B., Polini A., Azami Z., Leeuwenburgh S.C.G., Jansen J.A., Yang F., van den Beucken J.J.J.P. (2017). Incorporation of PLLA micro-fillers for mechanical reinforcement of calcium-phosphate cement. J. Mech. Behav. Biomed. Mater..

[45] Xie Z., Buschle-Diller G. (2011). Functionalized Poly(L-lactide) nanoparticles from electrospun nanofibers. J. Biomater. Sci. Polym. Ed..

[46] Polini A., Petre D.G., Iafisco M., de Lacerda Schickert S., Tampieri A., van den Beucken J., Leeuwenburgh S.C.G. (2017). Polyester fibers can be rendered calcium phosphate-binding by surface functionalization with bisphosphonate groups. J. Biomed. Mater. Res. A.

[47] Dulnik J., Denis P., Sajkiewicz P., Kołbuk D., Choińska E. (2016). Biodegradation of bicomponent PCL/gelatin and PCL/collagen nanofibers electrospun from alternative solvent system. Polym. Degrad. Stab..

[48] Kołbuk D., Guimond-Lischer S., Sajkiewicz P., Maniura-Weber K., Fortunato G. (2015). The Effect of Selected Electrospinning Parameters on Molecular Structure of Polycaprolactone Nanofibers. Int. J. Polym. Mater. Polym. Biomater..

[49] Li J., Xiao P., Li H., Zhang Y., Xue F., Luo B., Huang S., Shang Y., Wen H., de Claville C.J. (2015). Crystalline structures and crystallization behaviors of poly (L-lactide) in poly (L-lactide)/graphene nanosheet composites. Polym. Chem..

[50] Xu H., Zhong G.J., Fu Q., Lei J., Jiang W., Hsiao B.S., Li Z.M. (2012). Formation of shish-kebabs in injection-molded poly(L-lactic acid) by application of an intense flow field. ACS Appl. Mater. Interfaces.

[51] Flory P.J. (1945). Tensile Strength in Relation to Molecular Weight of High Polymers. J. Am. Chem. Soc..

[52] Bersted B.H., Anderson T.G. (1990). Influence of molecular weight and molecular weight distribution on the tensile properties of amorphous polymers. J. Appl. Polym. Sci..

[53] Yang Z., Zhengwei M., Huayu S., ChangYou G. (2012). In-depth study on aminolysis of poly(e-caprolactone): Back to the fundamentals. Sci. China Chem..

[54] Bech L., Meylheuc T., Lepoittevin B., Roger P. (2007). Chemical surface modification of poly (ethylene terephthalate) fibers by aminolysis and grafting of carbohydrates. J. Polym. Sci. Pol. Chem..

[55] Ganjaliniaa A., Akbaria S., Solouk A. (2017). PLLA scaffolds surface-engineered via poly(propylene imine) dendrimers for improvement on its biocompatibility/controlled pH biodegradability. Appl. Surf. Sci..

[56] Bakry A., Darwish M.S.A., El Naggar A.M.A. (2018). Assembling of hydrophilic and cytocompatible three-dimensional scaffolds based on aminolyzed poly(L-lactide) single crystals. New J. Chem..

[57] Antonova L.V., Seifalian A.M., Kutikhin A.G., Sevostyanova V.V., Krivkina E.O., Mironov A.V., Burago A.Y., Velikanova E.A., Matveeva V.G., Glushkova T.V. (2016). Bioabsorbable Bypass Grafts Biofunctionalised with RGD Have Enhanced Biophysical Properties and Endothelialisation Tested In vivo. Front. Pharmacol..

[58] Holmes S.A. (1996). Aminolysis of poly(ethylene terephthalate) in aqueous amine and amine vapour. J. Appl. Polym. Sci..

[59] Shimizu J., Okui N., Kikutani T., Ziabicki A., Kawai H. (1985). Fine Structure and Physical Properties of Fibers Melt-Spun at High-Speeds from Various Polymers. High-Speed Fiber Spinning, Science and Engineering Aspects.

[60] Shimizu J., Kikutani T., Takaku A. Structure Developments in High-Speed Spinning. Proceedings of the International Symposium Fiber Science and Technology.

[61] Perez G., Ziabicki A., Kawai H. (1985). Some effects of the rheological properties of polyethylene terephthalate) on spinning line profile and structure developed in high speed spinning. High-Speed Fiber Spinning, Science and Engineering Aspects.

[62] Perez G., Jung E. High Speed Melt Spinning: Fiber Structure and Properties. Proceedings of the International Symposium Fiber Science and Technology.

[63] Kameoka J., Craighead H.G. (2003). Fabrication of oriented polymeric nanofibers on planar surfaces by electrospinning. Appl. Phys. Lett..

[64] Kameoka J., Orth R., Yang Y., Czaplewski D., Mathers R., Coates G.W., Craighead H.G. (2003). A scanning tip electrospinning source for deposition of oriented nanofibers. Nanotechnology.

[65] Buer A., Ugbolue S.C., Warner S.B. (2001). Electrospinning and Properties of Some Nanofibers. Text. Res. J..

[66] Filatov Y., Budyk A., Kirichenko V. (2007). Connecticut, Electrospinning of Micro- and Nanofibers: Fundamentals and Applications in Separation and Filtration Process.

[67] Fennessey S.F., Farris R.J. (2004). Fabrication of aligned and molecularly oriented electrospun polyacrylonitrile nanofibers and the mechanical behaviour of their twisted yarns. Polymer.

[68] Behler K., Havel M., Gogotsi Y. (2007). New solvent for polyamides and its application to the electrospinning of polyamides 11 and 12. Polymer.

[69] Wang X., Cao J., Hu Z., Pan W., Liu Z. (2006). Jet Shaping Nanofibers and the Collection of Nanofiber Mats in Electrospinning. J. Mater. Sci. Technol..

[70] Reneker D.H., Yarin A.L. (2008). Electrospinning jets and polymer nanofibers. Polymer.

[71] Reneker D.H., Yarin A.L., Fong H., Koombhongse S. (2000). Bending instability of electrically charged liquid jets of polymer solutions in electrospinning. J. Appl. Phys..

[72] Wang C., Tsou S.Y., Lin H.S. (2012). Brill transition of nylon-6 in electrospun nanofibers. Colloid Polym. Sci..

[73] Piai J.F., da Silva M.A., Martins A., Torres A.B., Faria S., Reis R.L., Muniz E.C., Neves N.M. (2017). Chondroitin sulfate immobilization at the surface of electrospun nanofiber meshes for cartilage tissue regeneration approaches. Appl. Surf. Sci..

[74] Molisak-Tolwinska H., Wencel A., Figaszewski Z. (1998). The Effect of Hydrophilization of Polypropylene Membranes with Alcohols on Their Transport Properties. J. Macromol. Sci. A.

[75] Castro A.G.B., Yang F., van den Beucken J.J.J.P., Jansen J.A., Khang G. (2017). Handbook of Intelligent Scaffolds for Tissue Engineering and Regenerative.

[76] Shahidi S., Wiener J., Ghoranneviss M., Gunay M. (2013). Eco-Friendly Textile Dyeing and Finishing.

[77] Causa F., Battista E., Della M.R., Guarnieri D., Iannone M., Netti P.A. (2010). Surface Investigation on Biomimetic Materials to Control Cell Adhesion: The Case of RGD conjugation on PCL. Langmuir.

[78] Monnier A., Al Tawil E., Nguyen Q.T., Valleton J.M., Fatyeyeva K., Deschrevel B. (2018). Functionalization of poly (lactic acid) scaffold surface by aminolysis and hyaluronan immobilization: How it affects mesenchymal stem cell proliferation. Eur. Polym. J..

[79] Pavlinec J., Lazar M. (1995). Cross-linking of poly (methyl methacrylate) by aminolysis of ester functions with diamines. J. Appl. Polym. Sci.

